# Randomised controlled trial and economic evaluation of a targeted cancer awareness intervention for adults living in deprived areas of the UK

**DOI:** 10.1038/s41416-021-01524-5

**Published:** 2021-08-27

**Authors:** Yvonne Moriarty, Mandy Lau, Bernadette Sewell, Rob Trubey, Harriet Quinn-Scoggins, Sioned Owen, Louise Padgett, Vasiliki Kolovou, Julie Hepburn, Peter Buckle, Rebecca Playle, Julia Townson, Michael Robling, Stephanie Gilbert, Polyxeni Dimitropoulou, Adrian Edwards, Caroline Mitchell, Maura Matthews, Stephanie Smits, Fiona Wood, Richard D. Neal, Kate Brain, ABACus study team, Mari Jones, Angela Farr

**Affiliations:** 1 Centre for Trials Research, Cardiff University, Cardiff, UK; 2 Swansea Centre for Health Economics, College of Human and Health Sciences, Swansea University, Swansea, UK; 3 PRIME Centre Wales, Division of Population Medicine, School of Medicine, Cardiff University, Cardiff, UK; 4 Tenovus Cancer Care, Cardiff, UK; 5 Department of Health Sciences, University of York, York, UK; 6 School of Sport & Health Sciences, Cardiff Metropolitan University, Cardiff, UK; 7 Public Involvement Community, Health and Care Research Wales Support Centre, Cardiff, UK; 8 Marie Curie Research Voices, London, UK; 9 Academic Unit of Primary Medical Care, University of Sheffield, Northern General Hospital, Sheffield, UK; 10 Leeds Institute of Health Sciences, University of Leeds, Leeds, UK

**Keywords:** Cancer, Disease prevention, Randomized controlled trials

## Abstract

**Background:**

Cancer outcomes are poor in socioeconomically deprived communities, with low symptom awareness contributing to prolonged help-seeking and advanced disease. Targeted cancer awareness interventions require evaluation.

**Methods:**

This is a randomised controlled trial involving adults aged 40+ years recruited in community and healthcare settings in deprived areas of South Yorkshire and South-East Wales. Intervention: personalised behavioural advice facilitated by a trained lay advisor. Control: usual care. Follow-up at two weeks and six months post-randomisation. Primary outcome: total cancer symptom recognition score two weeks post-randomisation.

**Results:**

Two hundred and thirty-four participants were randomised. The difference in total symptom recognition at two weeks [adjusted mean difference (AMD) 0.6, 95% CI: −0.03, 1.17, *p* = 0.06] was not statistically significant. Intervention participants reported increased symptom recognition (AMD 0.8, 95% CI: 0.18, 1.37, *p* = 0.01) and earlier intended presentation (AMD −2.0, 95% CI: −3.02, −0.91, *p* < 0.001) at six months. “Lesser known” symptom recognition was higher in the intervention arm (2 weeks AMD 0.5, 95% CI: 0.03, 0.97 and six months AMD 0.7, 95% CI: 0.16, 1.17). Implementation cost per participant was £91.34, with no significant between-group differences in healthcare resource use post-intervention.

**Conclusions:**

Improved symptom recognition and earlier anticipated presentation occurred at longer-term follow-up. The ABACus Health Check is a viable low-cost intervention to increase cancer awareness in socioeconomically deprived communities.

**Clinical trial registration:**

ISRCTN16872545.

## Background

Inequalities in cancer survival exist in the United Kingdom [1, 2]. The deprivation gap in cancer outcomes partly reflects socioeconomic differences in stage at diagnosis [3], with prolonged time to symptom presentation contributing to later stage diagnosis in lower socioeconomic groups [4, 5]. Cancer risk behaviours including smoking, alcohol and poor diet also contribute to higher cancer incidence and mortality in deprived communities [6]. Since the inception of the UK National Awareness and Early Diagnosis Initiative [7] and International Cancer Benchmarking Partnership [8], efforts have been focussed on understanding the root causes of international variation in cancer outcomes, including later symptom presentation and later stage diagnosis.

Early symptoms of common cancers can be difficult to recognise, and low awareness of potential cancer symptoms has been linked to longer time to help-seeking [9, 10]. Previous studies have highlighted key behavioural influences on cancer symptom presentation, including perceived practical and emotional barriers [11], worry about wasting the doctor’s time [12] and fearful and fatalistic beliefs about cancer [13, 14], especially among adults living in deprived communities [15–17]. While evidence suggests that interventions delivered via mass media campaigns can improve cancer outcomes [18–20], they may not reach people from lower socioeconomic groups [21].

Innovative approaches are therefore needed to expedite cancer diagnosis and improve outcomes among people from lower socioeconomic groups who are disproportionately affected by cancer. Cancer awareness interventions that are targeted at social and community networks have the potential to engage people living in deprived areas by signalling that timely help-seeking for suspected cancer symptoms is welcomed and legitimised [22]. Building on personal connections and trust using the trained peer supporter model may be a powerful way of spreading positive cancer awareness messages through the community. This is an established idea, especially in areas of deprivation where long-term health may not be a priority due to low resources [23]. Evidence suggests that community-based behavioural interventions facilitated by trained and trusted peer supporters are important for engaging underserved populations in cancer awareness and normalising earlier help-seeking [24, 25].

We developed the Awareness and Beliefs About Cancer (ABACus) Health Check, a targeted and tailored intervention designed to raise awareness of common cancers and encourage timely symptom help-seeking among adults living in socioeconomically deprived communities [26]. Early evidence demonstrated successful engagement and potential for intervention scalability, reach and adoption in non-medical community settings in areas of deprivation [27]. We trialled the effectiveness of the intervention in increasing cancer awareness and help-seeking behaviour among adults recruited in socioeconomically deprived areas of Yorkshire and Wales.

## Methods

Trial methods are reported in detail in the published protocol [28].

### Study design

This was an unblinded, individually randomised controlled trial of a facilitated Health Check intervention hosted online and delivered by three trained lay advisors in areas of high deprivation in South and West Yorkshire and South-East Wales.

### Participants

Participant inclusion criteria were: adults aged ≥40 years recruited from venues in socioeconomically deprived areas (i.e. lowest quintile measured using the Index of Multiple Deprivation (IMD) [29]/Welsh Index of Multiple Deprivation (WIMD) [30]) of South and West Yorkshire (Sheffield, Barnsley, Rotherham, Wakefield, Doncaster) or South-East Wales (Merthyr Tydfil and Newport). Exclusion criteria were: non-English speaking, unable to give written informed consent, or participation in the Phase 2 study [28].

### Recruitment and settings

Full details of recruitment and settings for recruitment are published [27]. Briefly, local delivery organisations situated in eligible community and healthcare settings (i.e. located in an area of socioeconomic deprivation) were approached by the lay advisors, who sought permission to access the organisation’s facilities for recruitment purposes and delivering the Health Check intervention. They acted as recruitment venues and enabled access to members of the public and provided a suitable private space for the intervention delivery. Community settings included venues hosting local community events and groups, for example, job shops, libraries and sheltered housing. Healthcare settings included general practitioner (GP) surgeries and pharmacies.

### Sample size

We aimed to recruit 246 participants providing 90% power to detect an effect size of 0.5 in the primary outcome (using a two-sided *t* test, at the 5% significance level and assuming 30% attrition) to demonstrate an average increased recognition of one cancer symptom (SD = 2.2).

### Randomisation

Immediately following baseline data collection, participants were randomised to the intervention or control. Participants were randomised using permuted blocks of size 2, 4 and 6 on a 1:1 ratio and informed immediately of the outcome by the lay advisor.

### Intervention

The ABACus Health Check intervention was specifically developed to improve awareness of a range of cancers and reduce time to symptom presentation among adults living in areas of socioeconomic deprivation in the UK [22, 26]. The intervention was co-produced with local stakeholders and grounded in behaviour change theory, with social support and enablement provided by a credible lay advisor identified as key intervention functions [26]. In keeping with the principles of co-production, the intervention focussed on cancer symptom awareness, while acknowledging stakeholders’ views on the importance of addressing underlying cancer risk factors. It comprises an interactive touchscreen questionnaire about common cancer symptoms, cancer screening and risk factors (smoking, alcohol, diet and activity) with personalised results, support and advice delivered by a trained lay advisor drawing on seven behaviour change techniques (i.e. Information about health consequences; Prompts/cues; Credible source; Restructuring the social environment; Social support; Goal setting (behaviour) and Action planning) tailored to the individual’s results [22]. Three lay advisors were specifically employed to recruit participants and deliver the Health Check. They all had either undergraduate or Master’s level education and had a health promotion background. Further description of the intervention reported according to the TIDieR checklist [31] can be found in Supplementary Table 1.

### Control

Participants randomised to the control group were provided with the usual available care and support accessible through their GP or community organisations where applicable.

### Data collection

Data were collected at baseline, two weeks and six months following randomisation. At baseline, data were collected face-to-face using an iPad and entered directly into a bespoke study database. Subsequent data collection was carried out over the telephone or by postal survey according to the participant’s preference. Participants were offered a total of £15 in high street shopping vouchers as an incentive to take part, one supplied at baseline (£10) and one after completion of the six months follow-up questionnaire (£5).

### Process evaluation

The method and results of the process evaluation are reported separately (HQ-S, YM, SG, SS, VS, JH, et al. unpublished) and included participant interviews at two to six weeks post-baseline, audio recordings and observations of the Health Check delivery, interviews with the lay advisors prior to and post participant recruitment and recruitment day logs written by the lay advisors.

### Primary outcome

The primary outcome was total cancer symptom recognition score measured two weeks after randomisation, using an adapted version of the Awareness and Beliefs about Cancer measure (ABC) [32]. See Supplementary Table 2 for further details on measures used.

### Secondary outcomes

Secondary outcomes included anticipated symptom presentation for selected symptoms (unusual lump, rectal bleeding, persistent cough, unexplained weight loss); barriers to symptom presentation; beliefs about cancer; state anxiety (details of these in Supplementary Table 2); Health Check implementation costs; cost of subsequent healthcare resource use; demographic and health-related variables.

#### Health Check intervention implementation costs

Intervention costs considered in the health economic analysis included the cost of training the lay advisors, costs related to intervention face-to-face delivery and equipment to deliver the Health Check, which was assumed to be replaced after a mean lifespan of 3 years. Resource use and costs associated with intervention implementation were taken from recruitment day logs and notes, receipts and through discussions with the study team. Training costs were calculated based on data obtained from study training logs, costed using published unit costs [33]. ABACus Health Check development costs were not included in these costs as the intervention was fully developed [26] at the time of the study.

#### Cost of subsequent healthcare resource use

Subsequent healthcare resource use associated with symptoms that could be due to cancer at baseline and six months including primary care consultations, accident and emergency department visits, outpatient appointments, inpatient stays, imaging and investigations and advice related to potential cancer symptoms was established using a Client Service Receipt Inventory (CSRI) [34], adapted for healthcare resource use collection in people living in socioeconomically deprived. Each CSRI questionnaire asked for potential cancer symptom-related healthcare resource use in the past six months. Costs were assigned as 2018 Pound Sterling using published unit costs [33, 35]. Cancer treatment related to confirmed cancer was collected but not included in the total cost.

#### Demographic and health-related variables

Demographic background variables included age, gender, ethnicity, current relationship status, highest level of education, employment and home ownership. IMD [29] and WIMD [30] scores were calculated from postcodes. Experience of cancer (self, family and friends) and self-rated health were assessed.

### Statistical analyses

Data were analysed using intention-to-treat principles (i.e. participants remained in the groups to which they were assigned irrespective of intervention received) using complete cases. The primary analyses applied a linear model to total cancer symptom recognition score at two weeks follow-up. Mixed-effects two-level partial cluster regression models were used to adjust for lay advisors as a stratification variable and to allow for clustering by advisor. The distributional assumptions of the linear model were checked and transformed where appropriate. Bootstrapping was used to generate regression coefficients and confidence intervals (CIs) if the distributions remained non-normal. The mean (SD) score for the intervention and control groups at baseline and follow-up were tabulated. The primary outcome effect was presented adjusted for baseline score, with 95% CI. Pre-specified exploratory subgroup analyses were run for age, gender, recruitment setting and education. Secondary outcomes were analysed similarly. The consistency of conclusions drawn from the primary analysis was investigated by conducting sensitivity analyses for: (i) missing responses (i.e. not missing completely at random); (ii) data collected outside the designated two weeks follow-up window. Psychometric testing of baseline data was conducted to assess the internal validity and reliability of the adapted ABC measures, with Cronbach’s alpha coefficient to assess the internal consistency of the scales and appropriate regressions applied to subscales generated from factor analysis (Supplementary Table 3).

For all analyses, two-sided 95% CIs and *p* values were calculated. *p* Values < 0.05 were considered statistically significant. Statistical analyses were conducted using IBM SPSS version 25 and STATA version 15.

### Health economic analyses

The cost differences between intervention and control groups in the six months post-randomisation, based on all available cases, were calculated using SPSS version 26. Independent samples *t* tests, adjusted using Bonferroni–Holm sequential corrections [36], were used for comparison of control and intervention group data and paired *t* tests for within-group differences between baseline and six months follow-up, with a 5% significance level. A within-trial cost-effectiveness analysis calculated the incremental cost per point improvement in cancer symptom awareness score at the six months follow-up point in the intention-to-treat population. Trial results extrapolated to a longer time horizon and published evidence were used to estimate the incremental cost per quality-adjusted life-year (QALY) gained based on a combined decision tree and Markov model constructed using Microsoft Excel and Visual Basics for Applications over a five year time horizon. This analysis assumed that encouraging earlier presentation with cancer symptoms would result in potential effects on health outcomes and healthcare costs based on a simulated cohort of 100,000 people living in socioeconomically deprived areas of the UK. The analysis was undertaken from an NHS and personal social services perspective with costs and outcomes discounted at an annual rate of 3.5%. Deterministic and probabilistic sensitivity analyses were undertaken to test the robustness of the results.

## Results

**Fig. 1:**
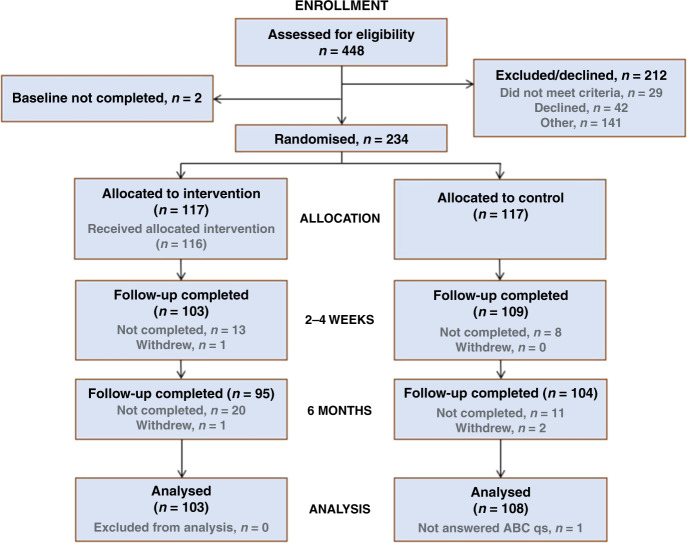
Consort flow diagram.

Overall, 448 members of the public were approached to take part during the study period between December 2017 and January 2019, of whom 29 were ineligible, 42 declined due to lack of interest and 141 declined for other reasons (e.g. insufficient time) (Supplementary Table 4). Following consent, two participants were withdrawn due to incomplete baseline questionnaires. A total of 234 participants (82 from South East Wales (35%) and 152 from South and West Yorkshire (65%)) were randomised to the intervention (*n* = 117, 50%) or control (*n* = 117, 50%) (Fig. 1). Despite a lower than planned sample recruited (*n* = 249), the study attrition rate of 9.5% [27] was much lower than anticipated (30%) [28] and therefore the trial was adequately powered.

Recruited participants had a mean age of 61.3 years (SD = 11.65). Most participants were white (229/234, 97.9%) and around two thirds were female (148/234, 63.2%). Around half of participants were retired (108/234, 46.2%) and had no formal qualifications or finished school at age ≤16 years (129/234, 55.1%). Around half of participants were living with a partner or spouse (123/234, 52.6%) and just over one-third were renting from a local authority or housing association (93/234, 39.7%). Most participants were resident in the 20% most deprived areas (WIMD: 49/82, 59.8%; IMD: 105/152, 69.1%). Baseline characteristics for participants (experiences of cancer) were well balanced between trial groups (Table 1). Baseline data were comparable between groups with a mean baseline cancer symptom recognition score of 9.0/12 and 8.9/12 in the control and Health Check groups, respectively (Supplementary Table 5). Retention was high at two weeks (212/234, 90.5%) and six months follow-up (99/234, 85.0%) and comparable between groups. No further sensitivity analyses were required (Supplementary Tables 6 and 7).

**Table 1:** Sociodemographic characteristics of participants at baseline (*N* = 234).

Characteristics	Control (*n* = 117)		Health Check (*n* = 117)		Total (*n* = 234)	
	*n*	%	*n*	%	*n*	%
Age (years), mean (SD)	61.9	(11.47)	60.7	(11.85)	61.3	(11.65)
Gender						
Male	50	42.7	36	30.8	86	36.8
Female	67	57.3	81	69.2	148	63.2
Highest level of education						
No qualifications/left school at 16 years	25	21.4	19	16.2	44	18.8
Finished school at or before age of 15 years	36	30.8	39	33.3	75	32.1
Completed GCSEs, O-Levels or equivalent	21	17.9	26	22.2	47	20.1
Completed A levels or equivalent	5	4.3	9	7.7	14	6.0
Completed further education but not degree	22	18.8	14	12.0	36	15.4
Completed a bachelor’s degree/masters/PhD	8	6.8	10	8.5	18	7.7
Employment status						
Employed full time	15	12.8	11	9.4	26	11.1
Employed part time	12	10.3	9	7.7	21	9.0
Full-time homemaker	1	0.9	2	1.7	3	1.3
Retired	58	49.6	50	42.7	108	46.2
Unemployed	15	12.8	22	18.8	37	15.8
Self-employed	3	2.6	3	2.6	6	2.6
Disabled or too ill to work	13	11.1	19	16.2	32	13.7
Prefer not to say	0	0.0	1	0.9	1	0.4
Home/living arrangement						
Own outright	48	41.0	34	29.1	82	35.0
Own mortgage	8	6.8	12	10.3	20	8.5
Rent form local authority/housing association	44	37.6	49	41.9	93	39.7
Rent privately	14	12.0	17	14.5	31	13.2
Living with family or friends	3	2.6	3	2.6	6	2.6
Prefer not to say	0	0.0	2	1.7	2	0.9
Current relationship						
Living with partner/spouse	63	53.8	60	51.3	123	52.6
Living alone (not living with partner/spouse)	51	43.6	52	44.4	103	44.0
Prefer not to say	3	2.6	5	4.3	8	3.4
Ethnic group						
White	115	98.3	114	97.4	229	97.9
White and Black Caribbean	0	0.0	1	0.9	1	0.4
White and Black African	1	0.9	0	0.0	1	0.4
Pakistani	1	0.9	0	0.0	1	0.4
Caribbean	0	0.0	1	0.9	1	0.4
Other ethnic group	0	0.0	1	0.9	1	0.4
Welsh Index of Multiple Deprivation (*n* = 82)						
10% most deprived	15	36.6	16	39.0	31	37.8
10–20% most deprived	9	22.0	9	22.0	18	22.0
20–30% most deprived	7	17.1	6	14.6	13	15.9
30–40% most deprived	6	14.6	3	7.3	9	11.0
50% least deprived	4	9.8	7	17.1	11	13.4
Index of Multiple Deprivation (*n* = 152)						
10% most deprived	47	61.8	37	48.7	84	55.3
20% most deprived	9	11.8	12	15.8	21	13.8
30% most deprived	3	3.9	8	10.5	11	7.2
40% most deprived	4	5.3	8	10.5	12	7.9
50% most deprived	2	2.6	0	0.0	2	1.3
50% least deprived	3	3.9	6	7.9	9	5.9
40% least deprived	1	1.3	2	2.6	3	2.0
30% least deprived	6	7.9	1	1.3	7	4.6
20% least deprived	1	1.3	1	1.3	2	1.3
10% least deprived	0	0.0	1	1.3	1	0.7
Have you had cancer?						
Yes	27	23.1	21	18.3	48	20.7
No	90	76.9	94	81.7	184	79.3
Has your partner had cancer?						
Yes	21	18.1	15	12.9	36	15.5
No	95	81.9	101	87.1	196	84.5
Has a close family member had cancer?						
Yes	83	70.9	93	79.5	176	75.2
No	34	29.1	24	20.5	58	24.8
Has another family member had cancer?						
Yes	65	55.6	61	53.0	126	54.3
No	52	44.4	54	47.0	106	45.7
Has a close friend had cancer?						
Yes	67	57.8	65	56.5	132	57.1
No	49	42.2	50	43.5	99	42.9
Has another friend had cancer?						
Yes	80	69.0	72	62.6	152	65.8
No	36	31.0	43	37.4	79	34.2

### Primary outcome

Difference in cancer symptom recognition score at two weeks follow-up was not statistically significant between groups (adjusted mean difference (AMD): 0.6, 95% CI: −0.03 to 1.17, *p* = 0.06) (Table 2). There was no evidence of an intervention effect in the pre-specified subgroup analyses and one ad hoc subgroup analysis (Supplementary Table 8). Primary outcome data collected outside the two weeks follow-up window were adjusted to include data collected outwith the two week window, with no change in difference (Supplementary Table 9).

**Table 2:** Primary and secondary outcome measures.

Outcome measures	Baseline				2 weeks follow-up				Unadjusted mean difference^a^	Adjusted mean difference^b^
	Control		Health Check		Control		Health Check			
	*n*	Mean (SD)	*n*	Mean (SD)	*n*	Mean (SD)	*n*	Mean (SD)	Difference (95% CI), *p* value	Difference (95% CI), *p* value
Cancer Symptom Recognition score (ABC)	117	9.0 (2.66)	117	8.9 (2.70	108	8.6 (3.05)	103	9.0 (2.89)	0.6 (−0.03, 1.17), 0.06	0.6 (−0.03, 1.17), 0.06
Anticipated Symptom Presentation score (ABC)	117	11.6 (5.32)	117	11.6 (5.45)	109	12.0 (5.17)	102	10.9 (4.49)	−0.8 (−1.79, 0.29), 0.16	−0.8 (−1.79, 0.29), 0.16
Barriers To Presentation score (ABC)^c^	117	1.9 (2.11)	116	2.3 (2.69)	107	1.8 (2.39)	103	2.5 (2.87)	0.1 (−0.17, 0.34), 0.51	0.1 (−0.23, 0.41), 0.60
Beliefs About Cancer score (ABC)	117	6.1 (1.76)	117	6.3 (2.00)	108	6.0 (2.00)	100	6.1 (2.29)	−0.02 (−0.50, 045), 0.92	−0.02 (−0.50, 0.45), 0.92
State Trait Anxiety Inventory score^d^	116	7.57 (3.30)	117	7.84 (3.13)	105	7.51 (3.24)	102	7.87 (3.73)	0.03 (−0.11, 0.18), 0.64	0.03 (−0.11, 0.18), 0.64

	Baseline				6 months follow-up					
	Control		Health Check		Control		Health Check			
	*n*	Mean (SD)	*n*	Mean (SD)	*n*	Mean (SD)	*n*	Mean (SD)		
Cancer Symptom Recognition score (ABC)	117	9.0 (2.66)	117	8.9 (2.70	104	8.9 (2.61)	94	9.7 (2.60)	0.8 (0.18, 1.37), 0.01	0.8 (0.18, 1.37), 0.01
Anticipated Symptom Presentation score (ABC)	117	11.6 (5.32)	117	11.6 (5.45)	104	12.2 (5.22)	95	9.8 (4.39)	−2.0 (−3.02, −0.90), <0.001	−2.0 (−3.02, −0.91), <0.001
Barriers To Presentation score (ABC)	117	1.9 (2.11)	116	2.3 (2.69)	103	1.8 (2.55)	92	2.1 (2.43)	0.3 (−0.29, 0.88), 0.32	0.3 (−0.36, 0.91), 0.39
Beliefs About Cancer score (ABC)	117	6.1 (1.76)	117	6.3 (2.00)	103	5.9 (1.97)	94	5.9 (1.78)	−0.03 (−0.45, 0.38), 0.88	−0.03 (−0.45, 0.38), 0.88
State Trait Anxiety Inventory score	116	7.57 (3.30)	117	7.84 (3.13)	103	7.84 (3.24)	94	8.77 (3.98)	0.81 (−0.10, 1.73), 0.08	0.81 (−0.10, 1.72), 0.08

^a^Single-level model adjusted for baseline score.

^b^Multilevel model adjusted for stratification (lay advisor) and baseline score.

^c^Zero-inflated negative binomial.

^d^Square root transformed.

### Secondary outcomes

At six months follow-up, participants allocated to the Health Check had statistically significantly increased symptom recognition (AMD: 0.8, 95% CI: 0.18 to 1.37, *p* = 0.01) and reported shorter anticipated time to presentation (AMD: −2.0, 95% CI: −3.02 to −0.91, *p* < 0.001) compared to control. There were no statistically significant differences between groups at two weeks in anticipated symptom presentation (AMD: −0.8, 95% CI: −1.79 to 0.29), perceived barriers to presentation (AMD: 0.1, 95% CI: −0.23 to 0.41), beliefs about cancer (AMD: −0.02, 95% CI: −0.50 to 0.45) and state anxiety (AMD: 0.03, 95% CI: −0.11 to 0.18). There was no evidence of difference between groups in perceived barriers (AMD: 0.3, 95% CI: −0.36 to 0.91), beliefs about cancer (AMD: −0.03, 95% CI: −0.45 to 0.38) and state anxiety (AMD: 0.81, 95% CI: −0.10 to 1.72) at six months (Table 2).

**Fig. 2:**
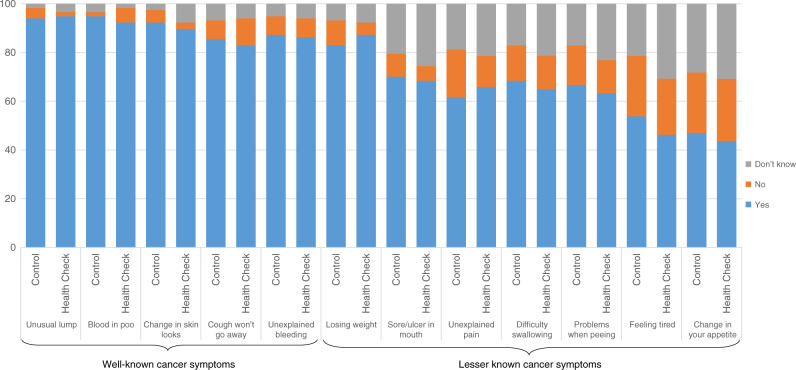
Percentage of participant responses for “well know” and “lesser known” cancer symptoms. Percentage of responses in cancer symptoms.

A large ceiling effect was observed for recognition of “well-known” cancer symptoms at baseline (Fig. 2). Health Check intervention participants had higher recognition of “lesser known” cancer symptoms at two weeks (AMD: 0.5, 95% CI: 0.03 to 0.97) and six months (AMD: 0.1, 95% CI: 0.16 to 1.17). No between-group difference was found for recognition of “well-known” cancer symptoms at two weeks (AMD: 0.1, 95% CI: −0.16 to 0.27) and six months (AMD: 0.1, 95% CI: −0.09 to 0.28) (Table 3).

**Table 3:** Regression analyses for sub-scores generated by factor analysis.

Outcome measures	Baseline				2 weeks follow-up				Adjusted mean difference^a^
	Control		Health Check		Control		Health Check		
	*n*	Mean (SD)	*n*	Mean (SD)	*n*	Mean (SD)	*n*	Mean (SD)	Difference (95% CI), *p* value
Cancer Symptom Recognition score (ABC) (7 items)	117	4.5 (2.17)	117	4.4 (2.13)	108	4.2 (2.37)	103	4.6 (2.20)	0.5 (0.03, 0.97), 0.04
Cancer Symptom Recognition score (ABC) (5 items)	117	4.5 (0.85)	117	4.5 (0.95)	109	4.4 (1.00)	103	4.4 (0.89)	0.1 (−0.16, 0.27), 0.61

	Baseline				6 months follow-up				
	Control		Health Check		Control		Health Check		
	*n*	Mean (SD)	*n*	Mean (SD)	*n*	Mean (SD)	*n*	Mean (SD)	
Cancer Symptom Recognition score (ABC) (7 items)	117	4.5 (2.17)	117	4.4 (2.13)	104	4.4 (2.17)	94	5.1 (2.15)	0.7 (0.16, 1.17), 0.01
Cancer Symptom Recognition score (ABC) (5 items)	117	4.5 (0.85)	117	4.5 (0.95)	104	4.5 (0.71)	95	4.6 (0.73)	0.1 (−0.09, 0.28), 0.31

^a^Adjusted for stratification (lay advisor) and baseline score.

### Health economics

During the study, 116 Health Checks were undertaken by three lay advisors at an intervention implementation cost of £91.34 per participant. This consisted of £12.51 lay advisor training cost, £21.05 staff cost for Health Check delivery and £57.78 equipment cost (including online hosting and technical support of the Health Check questionnaire, iPads, laptops and mobile phones and mobile printing equipment) per participant. Total healthcare costs related to symptoms that could be due to cancer (based on available cases) in the six months follow-up period included the cost of all primary and secondary care as well as diagnostic investigations and symptom-related advice (Table 4). Participants in the Health Check intervention group had £11.64 (95% CI: −£198.77 to £175.50) lower total healthcare costs compared to the control group in the six months after baseline. The difference was not statistically significant (*p* = 0.90). After adding the Health Check implementation costs for all participants in the Health Check intervention group who had available healthcare resource use data, participants in the intervention group accrued a mean cost of £225.82 (SD = £641.41) at six months. The difference of £79.70 (95% CI: −£107.43 to £266.84) compared to the control group was not statistically significant (*p* = 0.40).

**Table 4:** Mean per participant cost of healthcare resources (£) used in the 6 months post-randomisation (6-month follow-up measurements); all available cases.

Healthcare resource	Health Check group (*n* = 93)	Control group (*n* = 103)	Difference; 95% CI	*p* value
Primary care costs				
GP surgery visits (SD)	9.60 (39.83)	9.72 (27.81)	−0.12; −9.72 to 9.49	0.981
Home visits (SD)	0.00 (0.00)	0.63 (6.34)	−0.63; −1.92 to 0.67	0.343
Phone consultations (SD)	0.54 (3.17)	0.49 (3.01)	0.05; −0.82 to 0.92	0.905
NHS Direct phone call (SD)	0.00 (0.00)	0.00 (0.00)	n/a	n/a
Total cost of primary care use per participant (SD)	10.14 (40.65)	10.84 (29.83)	−0.69; −10.67 to 9.29	0.892
Secondary care costs				
A&E visits (SD)	1.94 (18.75)	3.11 (22.41)	−1.17; −7.02 to 4.69	0.695
Emergency admissions (SD)	22.22 (214.24)	5.01 (50.89)	17.20; −25.70 to 60.11	0.430
Ambulance call-outs (SD)	2.71 (26.12)	4.31 (43.75)	−1.60; −11.89 to 8.69	0.759
Outpatient visits (SD)	19.32 (63.13)	30.87 (119.23)	−11.55; −38.85 to 15.75	0.405
Elective inpatient stays (SD)	0.00 (0.00)	38.29 (388.60)	−38.29; −117.78 to 41.20	0.343
Total secondary care cost per participant (SD)	46.19 (278.54)	81.60 (551.27)	−35.40; −160.49 to 89.68	0.577
Investigation costs				
Diagnostic imaging (SD)	13.23 (44.61)	13.19 (47.89)	0.04; −13.04 to 13.12	0.995
Blood tests (SD)	6.98 (35.71)	15.76 (90.76)	−8.78; −28.60 to 11.04	0.383
Endoscopy (SD)	19.97 (78.12)	3.28 (23.41)	16.69; −0.01 to 33.39	0.050^a^
Biopsy (SD)	35.35 (224.03)	20.31 (86.39)	15.04; −31.94 to 62.01	0.529
Smear test (SD)	0.91 (8.78)	0.00 (0.00)	0.91; −0.90 to 2.72	0.320
Total investigation cost per participant (SD)	76.44 (350.79)	52.54 (188.19)	23.90; −54.37 to 102.17	0.548
Other costs				
Cancer advice (SD)	1.71 (7.51)	1.15 (6.13)	0.56; −1.37 to 2.48	0.568
Total cost of potential cancer investigation (SD)	134.48 (641.41)	146.12 (682.48)	−11.64; −198.77 to 175.50	0.903
Cancer treatment costs				
Radiotherapy (SD)	0.00 (0.00)	5.64 (57.29)	−5.64; −17.36 to 6.07	0.343
Chemotherapy (SD)	0.00 (0.00)	0.00 (0.00)	n/a	n/a
Surgery (SD)	0.00 (0.00)	146.03 (881.47)	−146.03; −318.31 to 26.24	0.096
Total cancer treatment cost per participant (SD)	0.00 (0.00)	151.68 (920.43)	−151.68; −331.57 to 28.21	0.098

*CI* confidence interval, *n* sample size, *SD* standard deviation, *n/a* not applicable.

^a^Result no longer statistically significant after Bonferroni–Holm correction for multiple comparisons.

Based on the differences in costs and outcomes in the intention-to-treat population, the Incremental Cost-Effectiveness Ratio (ICER) was calculated to be £108.85 per point improvement in cancer symptom recognition score, with ICERs ranging from £31.51 to £205.97 in the sensitivity analysis. When the incremental cost of Health Check implementation, changes in healthcare resource use and downstream changes in healthcare cost as well as survival due to potentially earlier cancer detection are considered, the model-based cost-utility analysis estimated that the intervention is on average £4.08 less costly and produces 0.005 more QALYs than the control and thus is dominating. No change to the base case conclusion was found in the one-way sensitivity analyses conducted. The Health Check was dominating or cost-effective in all analyses and produced marginal cost savings of £3.58 per person when no impact on early diagnosis probability was assumed. In 100,000 probabilistic sensitivity analysis iterations, use of the Health Check was found to be less costly and more effective than the control on average. However, due to the small differences in cost and effect, results are distributed across all four sectors of the cost-effectiveness plane (Supplementary Fig. 1). Overall, the probability that the Health Check is cost-effective at willingness-to-pay thresholds of £20,000 and £30,000 per QALY gained is 88.6 and 95.5%, respectively (Supplementary Fig. 2).

## Discussion

### Summary of key findings

We conducted a randomised controlled trial to evaluate the effectiveness of a novel targeted and tailored behaviour change intervention designed to improve cancer awareness and address barriers to symptom presentation among adults living in socioeconomically deprived communities of the UK. Differences in total symptom recognition were not observed at short-term follow-up, but there was evidence of longer-term knowledge retention and shorter intended presentation among participants who received the intervention. While baseline levels of symptom awareness were high, recognition of “lesser known” symptoms such as persistent fatigue and unexplained weight loss improved, and there was no evidence of increased anxiety due to taking part in the Health Check. Effects on perceived barriers to symptom presentation and beliefs about cancer were not observed. The Health Check intervention was delivered at low cost and did not generate additional healthcare resource use in the six months post intervention.

### Discussion of findings within context of the literature

The design and evaluation of cancer awareness interventions is challenging for several reasons. Intervention intensity and duration are often limited by financial constraints, and evaluation methods are typically restricted to observational designs with short follow-up (e.g. Ironmonger et al. [18], McCutchan et al. [20]). Furthermore, designing robust controlled trials of cancer awareness interventions is problematic due to the demands of selecting a suitable comparator and outcome measures, which may inadvertently compromise intervention design and dose. The Improving Rural Cancer Outcomes trial was unique in randomising whole communities in Western Australia to receive a symptom awareness campaign in intervention areas compared to a matched control area [37]. However, no significant intervention effects were observed, possibly reflecting the omission of TV broadcast media from the intervention due to concerns about cost and trial contamination. Personalised behaviour change interventions can aid in encouraging members of the public to engage with health services, as reported in a trial that assessed the effectiveness of a targeted GP postal letter to increase patient consultation rates for possible cancer symptoms. While an increase in the number of overall consultations among intervention arm participants was observed in this trial, the total number of patients with whom these took place did not increase [38]. Further targeted public health interventions are needed to engage those less likely to engage with cancer awareness initiatives and address the persistent socioeconomic disparity in cancer outcomes [39].

To our knowledge, the ABACus trial is the first to detail a complex behavioural cancer awareness intervention that successfully reached and engaged a highly deprived target population [27] and effected change in awareness and anticipated presentation for a range of common cancer symptoms. We used the validated Awareness and Beliefs about Cancer measure [32], adapted to low literacy levels and with additional measures of barriers derived from the Cancer Awareness Measure [40]. The finding of a baseline ceiling effect in participants’ recognition of potential cancer symptoms is suggestive of measurement reactivity [41–43] and may partly reflect the effect of completing baseline measures immediately prior to intervention delivery. This is similar to Hubbard et al. [44], who found subtle increases in cancer awareness among adolescents enrolled in a trial of a brief school-based cancer awareness intervention. Following participation in a cancer awareness roadshow, Smith et al. [45] reported increased awareness of cancer risk factors and help-seeking behaviour but not increased symptom knowledge, which they attributed to ceiling effects and low intervention dose. However, we found that symptom knowledge was retained six months post-intervention, suggesting that more intensive individually tailored and facilitated awareness interventions may be more effective for the target population over a longer period. Nevertheless, the development of guidance to support the minimisation of bias due to measurement reactions in studies of complex health interventions [46] is welcome and will improve future evaluation methods.

### Strengths and limitations

A key strength is that the intervention was developed based on the Medical Research Council guidelines for developing and evaluating complex interventions [47] and principles of co-production and was grounded in a theoretical understanding of the behavioural influences on cancer awareness and help-seeking in the target population [26, 48]. The trial was sufficiently powered to detect changes in symptom awareness and the process evaluation indicates that the ability to tailor the intervention to individual needs and the personalised, facilitated nature of its delivery most likely explain its positive impact on awareness and anticipated presentation (HQ-S, YM, SG, SS, VS, JH, et al. unpublished). However, it is unknown whether and to what extent control arm participants were exposed to other local or national cancer awareness campaigns and how these may have impacted on the findings.

We recognise the limitations of using self-report measures of knowledge, beliefs and behaviour and of measuring anticipated rather than actual symptom presentation behaviour, which may help to explain lack of observed effects. We acknowledge the low internal consistency of the adapted ABC cancer belief measure. The ABC measure was adapted to account for low literacy levels within our target population and we recognise that there is a need to further develop and test psychometrically robust measures of beliefs about cancer for use within this population group. Our sample also consisted of predominantly white British and native English speakers, further limiting the generalisability of the findings to a wider UK population including mixed ethnic groups and non-native English speakers who may present with additional needs not addressed by our intervention. Future UK-wide implementation of the Health Check would need to consider any adaptations required to accommodate the needs of these populations.

### Implication for policy/practice and research

The intervention has the potential to achieve public health benefits by encouraging cancer awareness and earlier presentation in socioeconomically deprived communities, with a lower cost than the median observed for public health interventions between 2011 and 2016 in the UK [49]. Our exploratory, model-based cost-utility analysis showed that the Health Check has the potential to be cost-effective, or even cost-saving, over the longer term by potentially reducing later stage diagnosis and therefore improving longer-term outcomes [50, 51]. Further prospective, longer-term follow-up of larger cohorts using routine service data would provide more confidence in the effects of the Health Check on delays in symptom presentation, cancer stage at diagnosis and cancer outcomes, all of which affect cost-effectiveness.

Non-specific symptoms [52] such as persistent fatigue and unexplained weight loss are particularly difficult for people to recognise and are more likely to be dismissed than classic “well-known” symptoms such as lumps and unexplained bleeding, because they can more easily be misattributed to the after effects of major illnesses, natural everyday occurrences such as minor illnesses, stress or the ageing process [9, 53, 54]. Individually tailored and demographically targeted community-based cancer awareness interventions such as the ABACus Health Check may complement the recently implemented Multidisciplinary/Rapid Diagnostic Centre referral pathways in England [55] and Wales [56] by increasing public awareness and prompting action in response to “lesser known”, common cancer symptoms that are predictive of earlier stage disease [51]. Such initiatives may be important in responding to the impact of the coronavirus disease 2019 pandemic on symptomatic presentation in primary care [57, 58].

Our model of trained peer supporters delivering cancer awareness messages may present future implementation challenges within the UK health service provision system. However, the findings of improved cancer symptom recognition over a longer period, particularly for non-specific symptoms, are encouraging. There is potential for this model to be embedded and integrated across primary care, third-sector organisations and large employers and to be further adapted to investigate how this approach can be used across diverse communities (i.e. ethnic minorities, non-English speakers). However, this would require significant adaptations to accommodate cultural sensitivities and language barriers and further investigation to identify potential obstacles for wide-scale implementation and normalisation within a diverse UK context.

### Summary

The ABACus Health Check is a viable low-cost intervention to increase cancer awareness and encourage earlier symptom presentation in socioeconomically deprived communities in the UK. Further implementation research is needed to collect longer-term presentation, diagnosis and outcome data and to evaluate whether the intervention can be scaled up and rolled out in the UK’s socioeconomically deprived communities.

## Supplementary information

Supplementary tables

CONSORT checklist

## Data Availability

De-identified participant data will be made available to the scientific community upon request with an agreed/signed data sharing agreement.
