# Supplementary material for: Randomised controlled trial and economic evaluation of a targeted cancer awareness intervention for adults living in deprived areas of the UK
Source: Br J Cancer. 2021 Aug 27;125(8):1100–10. doi: 10.1038/s41416-021-01524-5 (PMC8391006; doi:10.1038/s41416-021-01524-5)
Supplement: Supplementary file 1 — Supplementary tables [file 41416_2021_1524_MOESM1_ESM.docx]

**Supplementary Table 1: TIDieR checklist (Hoffmann et al (2014)*BMJ*2014;348:g1687 doi: 10.1136/bmj.g1687)**

| **TIDieR Item** | **Description** |
| --- | --- |
| Item 1: Brief name | RCT of a Health Check intervention delivered by a trained lay advisor and facilitated by an online questionnaire. |
| Item 2: Why | Early cancer symptom recognition and help-seeking can lead to earlier stage diagnosis, better treatment options and potentially improved long-term outcomes. Tailored behavioural interventions to improve symptom awareness have the potential to better reach people from low socioeconomic groups who are disproportionately affected by cancer. |
| Item 3: What (materials) | Tailored behaviour change advice delivered using an online Health Check questionnaire and in the context of a supportive conversation. An intervention manual provided details of all the information (i.e. all possible questions and result options colour coded (red, amber, green) behaviour change advice (tailored by the result) and associated Behaviour Change Technique for each response option) to be provided verbally to the participant by the lay advisor. |
| Item 4: What (procedures) | Participants completed the interactive touch screen questionnaire using a handheld tablet, on their own or with support from the lay advisor according to preference. The Health Check comprised 29 questions across three domains relating to cancer symptoms (‘About You’), cancer screening (‘Your lifestyle’) and cancer risk factors (‘Your Health’). Questions were filtered for relevance depending on the participant’s age and gender (i.e., cervical, bowel and breast screening). Individualised results were provided in the results section and displayed using a traffic light system. Tailored behaviour change advice and signposting information was then provided by the lay advisor as part of a supportive conversation. Seven behaviour change techniques were drawn upon, including: Information about health consequences; Prompts/cues; Credible source; Goal setting; Social support; Restructuring the social environment; Action planning. Individual results were printed on an A4 sheet and an action plan completed for participants to take away. |
| Item 5: Who provided | The intervention was delivered by one of three trained lay advisors. Lay advisors all had public health/health promotion backgrounds and were educated to undergraduate degree or postgraduate level. The lay advisors were specifically recruited to the role and fulfilled a dual role of participant recruitment and intervention delivery. All lay advisors received extensive training in both trial methods and recruitment procedures as well as intervention delivery and were required to pass a competency assessment (assessed by the Chief Investigator and a senior member of the research team) before being able to recruit and deliver the intervention. One of the lay advisors had previously been involved with the feasibility study and provided some of the training and supervision to the other less experienced advisors. |
| Item 6: How | The intervention was delivered face-to-face immediately following baseline assessment and randomisation. |
| Item 7: Where | The intervention was delivered in a private room in community (local libraries, community hubs, sheltered housing) and healthcare (GP practices, community pharmacies) settings. |
| Item 8: When and how much | 116 participants received the intervention. The intervention was delivered immediately following baseline data collection and randomisation. Intervention duration lasted between 19-75 minutes with average delivery taking 30 minutes. Variation in length was attributed to interruptions, technical issues, and personal characteristics of participants. In theory a maximum of 89 Behaviour Change Techniques (BCT) could be delivered to an individual participant, although the actual number of BCTs delivered varied by participant depending on their responses to the Health Check questions. |
| Item 9: Tailoring | Behaviour Change Techniques delivered to each participant were tailored depending on participants’ responses to the Health Check questionnaire. Where participants’ responses flagged in ‘green’, these indicated areas where no change/signposting was required and no information was required to be delivered Responses in ‘amber’ were areas where change could be considered and all information and BCTs were to be delivered, and responses in ‘red’ were areas indicating where action should be taken and all relevant information and BCTs were to be delivered. |
| Item 10: Modifications | No modifications to the intervention took place during the trial. |
| Item 11: How well (planned) | To assess level of compliance with intervention delivery, 20% of intervention sessions were due to be audio-recorded and observed. This would enable ascertainment of intervention delivery according to manualised BCTs. A structured coding framework following the manualised BCTs was used to assess fidelity. |
| Item 12: How well (actual) | Fidelity of delivery was assessed through audio-recordings (10%, n =12) of intervention delivery sessions, of which 7 were additionally observed (n=7) across the three lay advisors. |

**Supplementary Table 2: Measures summary**

| **Domain** | **Measure** | **Assessment point** | **Question** | **Response options** | **Scoring/Analysis** |
| --- | --- | --- | --- | --- | --- |
| **PRIMARY ANALYSIS** | | | | | |
| Cancer symptom recognition | 12-item (e.g. ‘a cough that won’t go away’, ‘a sore or ulcer in your mouth that will not heal’, ‘difficulty swallowing’) score adapted from the validated ABC^1^ | Baseline,  2 weeks,  6 months | “The following may or may not be warning signs for cancer. We are interested in your opinion. Please tell us if you think the following are warning signs of cancer”. | 1. ‘Yes’ 2. ‘No’ 3. ‘Don’t know’ | ‘No’ and ‘Don’t know’ responses were combined.  The number of individual symptoms correctly recognised was calculated to create an aggregated symptom recognition score ranging from 0 to 12, with a higher score indicating higher symptom recognition.    For the purposes of secondary analysis factor analysis was carried out and the two factor-derived subscales were labelled “well-known symptoms” (five items) and “lesser known” symptoms (seven items) (see Supplementary Table 3) |
| **SECONDARY ANALYSIS** | | | | | |
| Anticipated symptom presentation | 4 items adapted from the ABC^1^, assessing anticipated time to presenting with “well known” symptoms (‘blood in your poo’, ‘an unusual lump’) and ‘“lesser known” symptoms (‘a cough that won’t go away’, ‘losing weight without trying to’). | Baseline,  2 weeks,  6 months | “For each of the signs and symptoms below, please tell us how long it would take you to go to the doctor from the time you first noticed the symptom”. | 1. ‘I would go as soon as I noticed’ 2. ‘Up to 1 week’ 3. ‘Over 1 up to 2 weeks’ 4. ‘Over 2 up to 3 weeks’ 5. ‘Over 3 up to 4 weeks’ 6. ‘More than a month’ 7. ‘I would not contact my doctor’ | Total score ranged from 4-28 (Cronbach’s alpha=0.74) with a higher score indicating longer time to presentation. |
| Barriers to symptom presentation | 7 items adapted from the ABC^1^ and Cancer Awareness Measure (CAM)^2^. | Baseline,  2 weeks,  6 months | “Sometimes people put off going to see the doctor even when they have a symptom they think might be serious. Could you say if any of these might put you off going to the doctor?”    Four emotional barriers (e.g. “I would be too scared”), one practical barrier (“It would be difficult for me to arrange transport to the doctor’s surgery”) and two service barriers (e.g. “My doctor would be difficult to talk to”). | 1. ‘Yes, often’ 2. ‘Yes, sometimes’ 3. ‘No’ | Response options were recoded as yes or no and summed to create a total perceived barriers score ranging from 0-14 (Cronbach’s alpha=0.70). |
| Beliefs about cancer | 4-items adapted from the ABC^1^. | Baseline,  2 weeks,  6 months | “For each of the statements below can you tell us how much you agree or disagree with each item”.     - “I would not want to know if I had cancer” - “I think that cancer can often be cured” - “I think that going to the doctor as quickly as possible after noticing a symptom of cancer could increase the chances of surviving” - “I think that a diagnosis of cancer is a death sentence”. | 1. ‘Strongly agree’ 2. ‘Tend to agree’ 3. ‘Tend to disagree’ 4. ‘Strongly disagree’ | Total cancer beliefs score ranged from 4-16 (Cronbach’s alpha=0.40) with a higher score denoting more negative beliefs. |
| State anxiety | 6-item short form of the validated State Trait Anxiety Inventory (STAI)^3^ | Baseline,  2 weeks,  6 months | “For each statement below select the most appropriate statement to tell us how you feel right now, at this moment:”     - “I feel calm” - “I am tense” - “I feel upset” - “I am relaxed” - “I feel content” - “I am worried” | 1. ‘Not at all’ 2. ‘Somewhat’ 3. ‘Moderately’ 4. ‘Very much’ | Total score ranged between 6-24 (Cronbach’s alpha=0.81) with a higher score indicating higher state anxiety. |

1. Simon AE, Forbes LJL, Boniface D, Warburton F, Brain KE, Dessaix A, et al. An international measure of awareness and beliefs about cancer: Development and testing of the ABC. BMJ Open. 2012;2(e001758).

2. Stubbings S, Robb K, Waller J, Ramirez A, Austoker J, Macleod U, et al. Development of a measurement tool to assess public awareness of cancer. Br J Cancer. 2009;101:S13–7.

3. Marteau TM, Bekker H. The development of a six‐item short‐form of the state scale of the Spielberger State—Trait Anxiety Inventory (STAI). Br J Clin Psychol. 1992;31:301–6.

**Supplementary Table 3: Factor analysis**

| **Cancer symptom recognition scale** | **Less well-known factor loadings** | **Well known factor loadings** |
| --- | --- | --- |
| A cough that won’t go away |  | 0.557 |
| An unusual lump |  | 0.675 |
| A change in how your skin looks (e.g. a change to a mole, freckle or patch of skin) |  | 0.559 |
| A change in your poo or any blood in your poo (e.g. having looser poo, a change in how often you go, or difficulty going) |  | 0.581 |
| Unexplained bleeding (e.g. blood in your pee, bleeding from your bottom, vaginal bleeding during /after sex or in between periods?) |  | 0.582 |
| A sore or ulcer in your mouth that will not heal | 0.665 |  |
| Problems when peeing (e.g. having to get up in the night to go, pain when peeing or problems with the flow) | 0.593 |  |
| Difficulty swallowing | 0.571 |  |
| Losing weight without trying to | 0.514 |  |
| Unexplained change in your appetite | 0.792 |  |
| Feeling tired most of the time | 0.700 |  |
| Unexplained pain that wont go away | 0.591 |  |

Extraction method: Principal component analysis

**Supplementary Table 4: Reasons for non-participation**

| **Reason** | **Number** | **Further detail** |
| --- | --- | --- |
| Ineligible for inclusion | 29 | Under 40 years (12) |
|  |  | Took part in phase 2 (7) |
|  |  | Non-English speaker (4) |
|  |  | Does not live in a socioeconomically deprived area (4) |
|  |  | Unable to provide written informed consent read and write (2) |
| Declined to participate  (not interested) | 42 |  |
| Other reasons | 141 | Did not have time (62) |
|  |  | At site to participate in a class only (15) |
|  |  | Had to leave to catch a bus (4) |
|  |  | Unwell (10) |
|  |  | Had an appointment to go to (4) |
|  |  | Adverse weather conditions (could not attend pre-booked appointment) (5) |
|  |  | Expressed an interest, said they would return later, did not do so (6) |
|  |  | Going on holiday after doctors’ appointment (1) |
|  |  | Had to return to work after doctors appointment (7) |
|  |  | Had to go and collect Grandchild (3) |
|  |  | Cancelled pre-booked health check appointment (4) |
|  |  | No-show for pre-booked appointment (3) |
|  |  | Waiting for a phone call (1) |
|  |  | Does not like talking about cancer and does not want to know any more about it (1) |
|  |  | Carer for spouse so had to return home after appointment (1) |
|  |  | Had family visiting (1) |
|  |  | Had to collect child from school (1) |
|  |  | Waiting for a GP appointment (2) |
|  |  | Hard of hearing so would struggle to take part (1) |
|  |  | Did not provide reason (6) |
|  |  | Enrolled in another research study (1) |
|  |  | Waiting for someone (1) |
|  |  | Recently lost someone to cancer so did not want to talk about it (1) |
| **TOTAL** | **212** |  |

**Supplementary Table 5: Baseline outcome measures (N=234)**

| **Variable** | **Control** | | **Health Check** | |
| --- | --- | --- | --- | --- |
|  | **n** | **Mean (SD)** | **n** | **Mean (SD)** |
| Cancer Symptom Recognition score | 117 | 9.0 (2.66) | 117 | 8.9 (2.70) |
| Anticipated Symptom Presentation | 117 | 11.6 (5.32) | 117 | 11.6 (5.45) |
| Barriers To Presentation | 117 | 1.9 (2.11) | 116 | 2.3 (2.69) |
| Beliefs About Cancer score | 117 | 6.1 (1.76) | 117 | 6.3 (2.0) |
| State Trait Anxiety Inventory score | 116 | 7.57 (3.30) | 117 | 7.84(3.13) |

**Supplementary Table 6: Trial allocation and sociodemographic characteristics of participants with and missing two weeks data (N=234)**

| **Variables** | **Missing two weeks data (n=23)** | | **With two weeks data (n=211)** | |
| --- | --- | --- | --- | --- |
|  | **n** | **%** | **n** | **%** |
| Trial arm |  |  |  |  |
| Control | 8 | 7.7 | 109 | 92.3 |
| Heath Check | 14 | 12.0 | 103 | 88.0 |
| Recruitment site |  |  |  |  |
| South Wales | 10 | 12.2 | 72 | 87.8 |
| South West Yorkshire | 13 | 8.6 | 139 | 91.5 |
| Recruitment setting |  |  |  |  |
| Community setting | 19 | 10.8 | 157 | 89.2 |
| Healthcare setting | 4 | 6.9 | 54 | 93.1 |
| Age (year) Mean (SD) | 54.7 (2.91) | | 62.0 (0.77) | |
| Gender |  |  |  |  |
| Male | 9 | 10.5 | 77 | 89.5 |
| Female | 14 | 9.5 | 134 | 90.5 |
| Highest level of education* |  |  |  |  |
| Finished school before age of 16 | 12 | 10.1 | 107 | 89.9 |
| Finished school after age 16 | 111 | 9.6 | 104 | 90.4 |
| Self-rated health |  |  |  |  |
| Good | 18 | 9.0 | 183 | 91.0 |
| Poor | 4 | 12.9 | 27 | 87.1 |
| Employment status |  |  |  |  |
| Employed full-time | 5 | 19.2 | 21 | 80.8 |
| Employed part-time | 3 | 14.3 | 18 | 85.7 |
| Full-time homemaker | 0 | 0 | 3 | 100.0 |
| Retired | 5 | 4.6 | 103 | 95.4 |
| Unemployed | 4 | 10.8 | 33 | 89.2 |
| Self-employed | 0 | 0.0 | 6 | 100.0 |
| Disabled or too ill to work | 6 | 18.8 | 26 | 81.3 |
| Prefer not to say | 0 | 0 | 1 | 100.0 |
| Welsh index of multiple deprivation (n=82) |  |  |  |  |
| 10% most deprived | 7 | 22.6 | 24 | 77.4 |
| 10 – 20% most deprived | 1 | 5.6 | 17 | 94.4 |
| 20 – 30% most deprived | 1 | 7.7 | 12 | 92.3 |
| 30 - 40% most deprived | 0 | 0.0 | 9 | 100.0 |
| 50% least deprived | 1 | 9.1 | 10 | 90.9 |
| Index of Multiple Deprivation (n=152) |  |  |  |  |
| 10% most deprived | 9 | 10.7 | 75 | 89.3 |
| 20% most deprived | 2 | 9.5 | 19 | 90.5 |
| 30% most deprived | 1 | 9.1 | 10 | 90.9 |
| 40% most deprived | 0 | 0.0 | 12 | 100.0 |
| 50% most deprived | 0 | 0.0 | 2 | 100.0 |
| 50% least deprived | 1 | 11.1 | 8 | 88.9 |
| 40% least deprived | 0 | 0.0 | 3 | 100.0 |
| 30% least deprived | 0 | 0.0 | 7 | 100.0 |
| 20% least deprived | 0 | 0.0. | 2 | 100.0 |
| 10% least deprived | 0 | 0.0 | 1 | 100.0 |

*Finished school before age of 16 including no qualifications or left school at 16, and finished school at or before age of 15; Finished school after age 16 including completed GCSEs, O-Levels or equivalent, completed A levels or equivalent, completed further education but not degree, and completed a bachelor’s degree/masters/PhD.

**Supplementary Table 7: Trial allocation and sociodemographic characteristics of participants with and missing six months data (N=234)**

| **Variables** | **Missing six months data (n=36)** | | **With six months data (n=198)** | |
| --- | --- | --- | --- | --- |
|  | **n** | **%** | **n** | **%** |
| Trial arm |  |  |  |  |
| Control | 13 | 11.1 | 104 | 88.9 |
| Health Check | 23 | 19.7 | 95 | 90.3 |
| Recruitment site |  |  |  |  |
| South Wales | 16 | 19.5 | 66 | 80.5 |
| South West Yorkshire | 20 | 13.2 | 132 | 86.8 |
| Recruitment setting |  |  |  |  |
| Community setting | 20 | 11.4 | 156 | 88.6 |
| Healthcare setting | 16 | 27.6 | 42 | 72.4 |
| Age (year) Mean (SD) | 56.1 (2.13) | | 62.2 (0.80) | |
| Gender |  |  |  |  |
| Male | 19 | 22.1 | 67 | 77.9 |
| Female | 17 | 11.5 | 131 | 88.5 |
| Highest level of education* |  |  |  |  |
| Finished school before age of 16 | 22 | 18.5 | 97 | 81.5 |
| Finished school after age 16 | 14 | 12.2 | 101 | 87.8 |
| Self-rated health |  |  |  |  |
| Good | 29 | 14.4 | 172 | 85.6 |
| Poor | 6 | 19.4 | 25 | 80.7 |
| Employment status |  |  |  |  |
| Employed full-time | 6 | 23.1 | 20 | 76.9 |
| Employed part-time | 2 | 9.5 | 19 | 90.5 |
| Full-time homemaker | 0 | 0.0 | 3 | 100.0 |
| Retired | 9 | 8.3 | 99 | 91.7 |
| Unemployed | 9 | 24.3 | 28 | 75.7 |
| Self-employed | 1 | 16.7 | 5 | 83.3 |
| Disabled or too ill to work | 9 | 28.1 | 23 | 71.9 |
| Prefer not to say | 0 | 0.0 | 1 | 100.0 |
| Welsh index of multiple deprivation (n=82) |  |  |  |  |
| 10% most deprived | 11 | 35.5 | 20 | 64.5 |
| 10 – 20% most deprived | 1 | 5.6 | 17 | 94.4 |
| 20 – 30% most deprived | 1 | 7.7 | 12 | 92.3 |
| 30 - 40% most deprived | 2 | 22.2 | 8 | 77.8 |
| 50% least deprived | 1 | 9.1 | 10 | 90.9 |
| Index of Multiple Deprivation (n=152) |  |  |  |  |
| 10% most deprived | 13 | 15.5 | 71 | 84.5 |
| 20% most deprived | 2 | 9.5 | 19 | 90.5 |
| 30% most deprived | 4 | 36.4 | 7 | 63.6 |
| 40% most deprived | 0 | 0.0 | 12 | 100.0 |
| 50% most deprived | 0 | 0.0 | 2 | 100.0 |
| 50% least deprived | 0 | 0.0 | 9 | 100.0 |
| 40% least deprived | 1 | 33.3 | 2 | 66.7 |
| 30% least deprived | 0 | 0.0 | 7 | 100.0 |
| 20% least deprived | 0 | 0.0 | 2 | 100.0 |
| 10% least deprived | 0 | 0.0 | 1 | 100.0 |

*Finished school before age of 16 including no qualifications or left school at 16, and finished school at or before age of 15; Finished school after age 16 including completed GCSEs, O-Levels or equivalent, completed A levels or equivalent, completed further education but not degree, and completed a bachelor’s degree/masters/PhD.

**Supplementary Table 8: Subgroup analysis for primary outcome measure**

We investigated differential intervention effects on primary outcome for five pre-specified subgroups and one ad-hoc subgroup by extending out regression models to include a sub-group by trial arm interaction. The pre-specified sub-groups of interest were age of participant; sex of participant (female, male); self-rated health (Good, poor); highest education level (finished school before age of 16, finished school after age 16); recruitment setting (community setting, healthcare setting). The ad-hoc subgroup was level of deprivation (20% most deprived or below, above 20% most deprived).

| Subgroup analysis | Variable | Adjusted mean difference* (95% CI) | P-value |
| --- | --- | --- | --- |
| Age of participant (n=211) | Control | Reference category for trial arm main effect | 0.27 |
|  | Health Check | 1.9 (-1.46, 5.29) |  |
|  | Age of participant (per year increase) | -0.01 (-0.05, 0.03) | 0.60 |
|  | Control x Age | Reference category for trial arm x age | 0.42 |
|  | Health Check x Age | -0.2 (-0.08, 0.03) |  |
| Subgroup analysis | Variable | Adjusted mean difference* (95% CI) | P-value |
| Sex of participant (n=211) | Control | Reference category for trial arm main effect | 0.78 |
|  | Health Check | -0.1 (-1.15, 0.86) |  |
|  | Male | Reference category for gender main effect | 0.22 |
|  | Female | -0.5 (-1.37, 0.31) |  |
|  | Control x Male | Reference category for trial arm x gender | 0.08 |
|  | Health Check x Female | 1.1 (-0.14, 2.37) |  |
| Subgroup analysis | Variable | Adjusted mean difference* (95% CI) | P-value |
| Self-rated health (n=210) | Control | Reference category for trial arm main effect |  |
|  | Health Check | 0.4 (-0.28, 1.00) | 0.27 |
|  | Good health | Reference category for self-rated health main effect |  |
|  | Poor health | -0.7 (-1.94, 0.46) | 0.23 |
|  | Control x Good health | Reference category for trial arm x self-rated health |  |
|  | Health Check x Poor health | 1.4 (-0.39, 3.19) | 0.13 |
| Subgroup analysis | Variable | Adjusted mean difference* (95% CI) | P-value |
| Highest education level (n=211) | Control | Reference category for trial arm main effect | 0.45 |
|  | Health Check | 0.3 (-0.50, 1.14) |  |
|  | Finished school before age of 16 | Reference category for highest education level main effect | 0.11 |
|  | Finished school after age 16 | 0.7 (-0.15, 1.51) |  |
|  | Control x Before age of 16 | Reference category for trial arm x highest education level | 0.44 |
|  | Health Check x Age 16 or above | 0.5 (-0.71, 1.64) |  |
| Subgroup analysis | Variable | Adjusted mean difference* (95% CI) | P-value |
| Recruitment setting (n=211) | Control | Reference category for trial arm main effect | 0.21 |
|  | Health Check | 0.5 (-0.25, 1.14) |  |
|  | Community | Reference category for recruitment setting main effect | 0.25 |
|  | Healthcare | -0.6 (-1.55, 0.40) |  |
|  | Control x Community | Reference category for trial arm x recruitment setting | 0.46 |
|  | Health Check x Healthcare | 0.5 (-0.86, 1.89) |  |
| Subgroup analysis | Variable | Adjusted mean difference* (95% CI) | P-value |
| Welsh index of multiple deprivation (WIMD)† (n=72) | Control | Reference category for trial arm main effect | 0.28 |
|  | Health Check | 0.8 (-0.69, 2.33) |  |
|  | 20% most deprived | Reference category for deprivation main effect | 0.65 |
|  | Above 20% most deprived to 50% least deprived | 0.4 (-1.26, 2.01) |  |
|  | 20% most deprived X Community | Reference category for trial arm x deprivation | 0.49 |
|  | Above 20% most deprived to 50% least deprived x Healthcare | -0.8 (-3.09, 1.51) |  |
| Subgroup analysis | Variable | Adjusted mean difference* (95% CI) | P-value |
| Index of multiple deprivation (IMD)‡ (n=139) | Control | Reference category for trial arm main effect | 0.11 |
|  | Health Check | 0.7 (-0.15, 1.57) |  |
|  | 20% most deprived | Reference category for deprivation main effect | 0.05 |
|  | 30% most deprived to 10% least deprived | 1.1 (0.02, 2.22) |  |
|  | 20% most deprived X Community | Reference category for trial arm x deprivation | 0.43 |
|  | 30% most deprived to 10% least deprived x Healthcare | -0.6 (-2.14, 0.91) |  |

*Adjusted for stratification (lay advisor) and baseline score; †20% most deprived or below category included 10% most deprived, and 10% to 20% most deprived categories from WIMD; Above 20% most deprived to 50% least deprived category included 20% to 30% most deprived, 30% to 40% most deprived, and 50% least deprived categories; ‡20% most deprived category include 10% most deprived and 20% most deprived categories from IMD; 30% most deprived to 10% least deprived category included 30% most deprived, 40% most deprived, 50% most deprived, 50% least deprived, 40% least deprived, 30% least deprived, 20% least deprived, and 10% least deprived categories.

**Supplementary Table 9: Primary outcome measure adjusted for data collection window**

The two-week follow-up data collection window was defined as days between 14 and 28. Two models were fitted; the first model included the data collection window as a binary covariate (within 28 days, after 28 days), and the second model included the data collection as a numerical covariate. Both models show that there is no effect of data collection out with the 2-week window.

| **Primary outcome data collection** | **Control (n=109)** | | **Health check (n=103)** | | **Adjusted mean difference* (95% CI), p-value** |
| --- | --- | --- | --- | --- | --- |
|  | **n** | **%** | **n** | **%** |  |
| Within 28 days (n, %) | 102 | 93.6 | 96 | 93.2 |  |
| After 28 days (n, %) | 7 | 6.4 | 7 | 6.8 | 0.6 (-0.002, 1.21), 0.05 |
| Data collection days mean (SD) | 18.9 (7.97) | | 19.8 (6.77) | | 0.6 (0.001, 1.21), 0.05 |

*Adjusted for stratification (lay advisor) and baseline score

Supplementary Figure 1: Cost-effectiveness plane illustrating the distribution of incremental cost per QALY gained over 100,000 analysis iterations following parameter resampling within pre-defined ranges and distributions based on a simulation cohort of 100,000 patients.


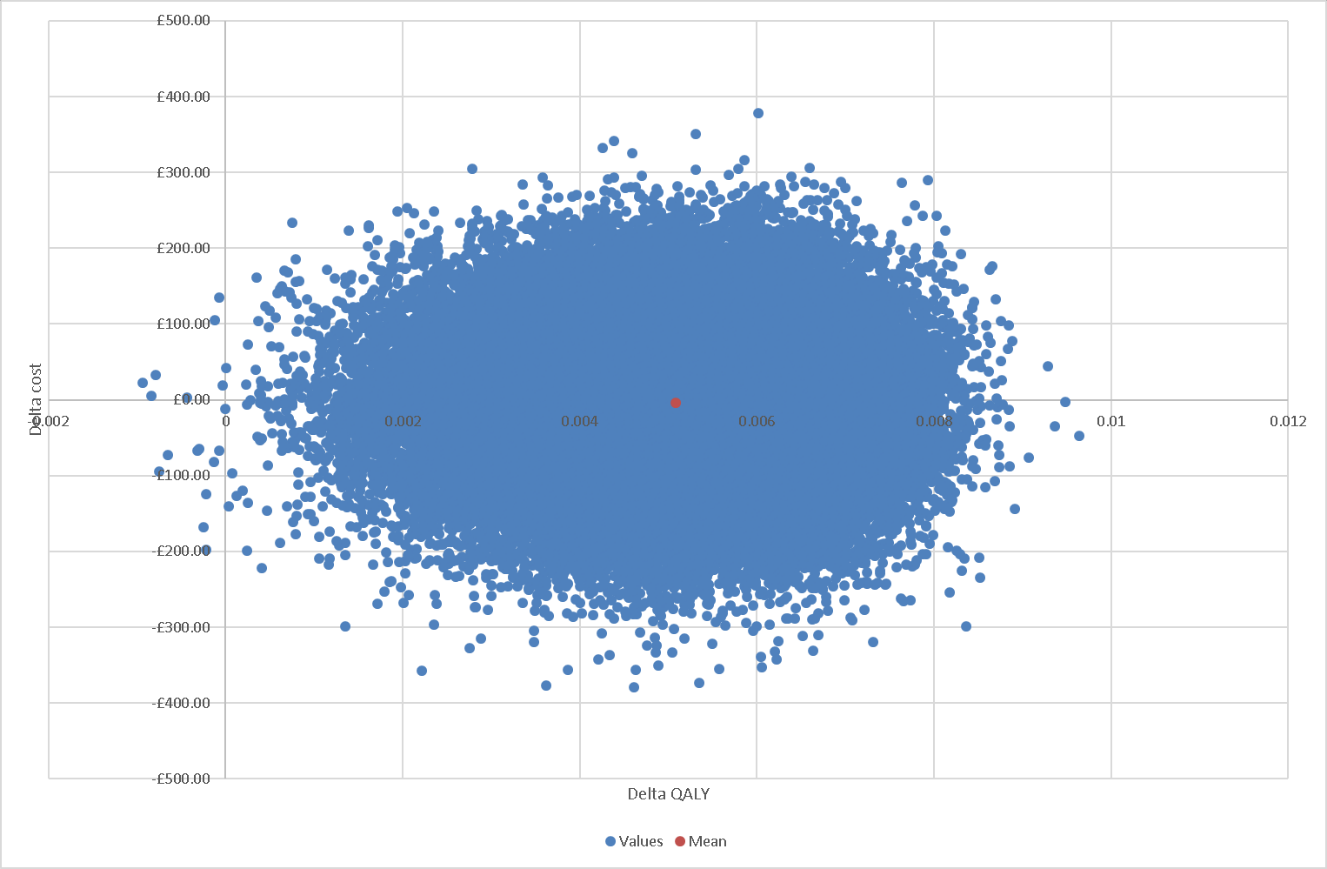


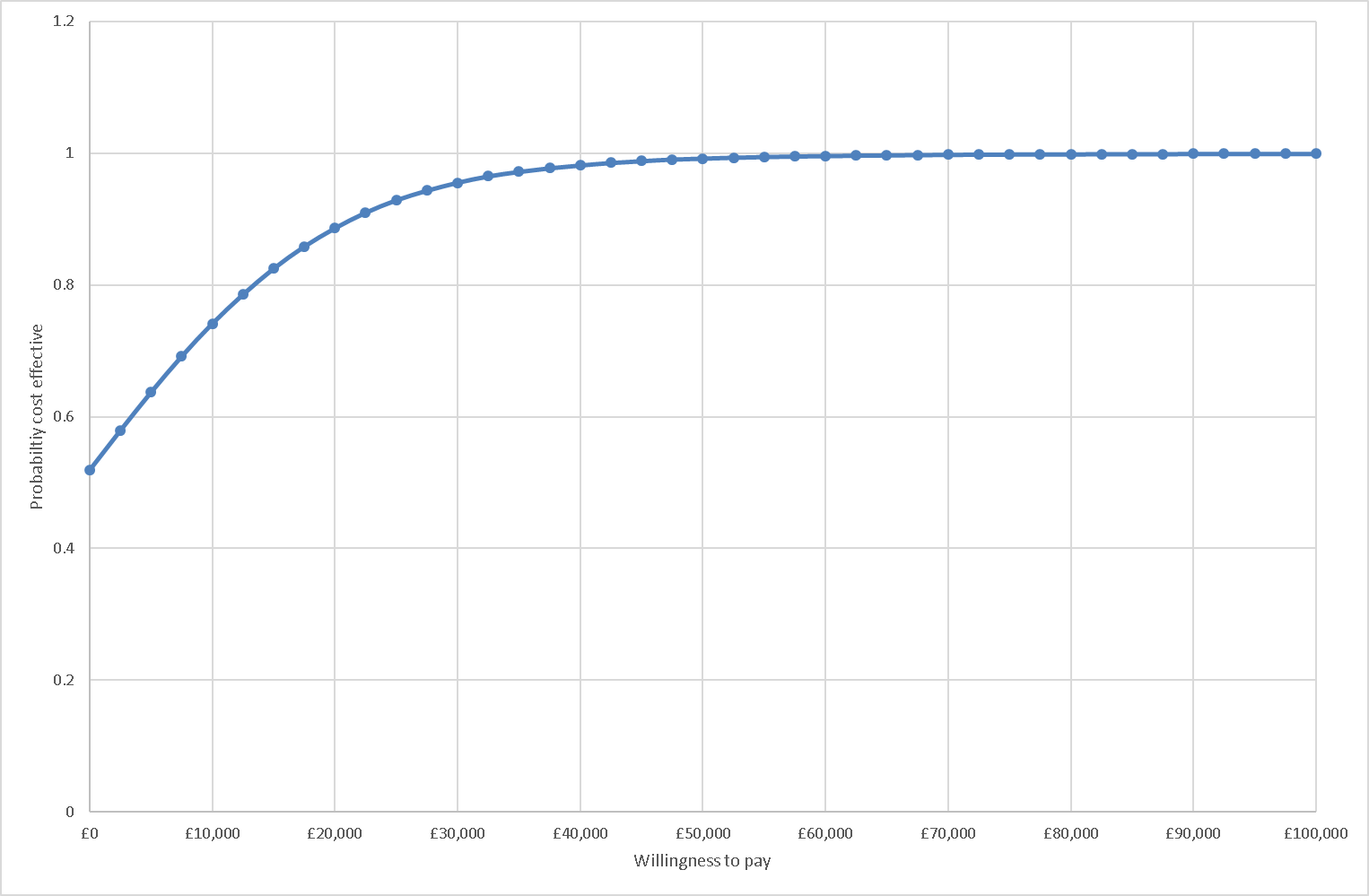
Supplementary Figure 2: Cost-effectiveness acceptability curve depicting the probability of the health check being cost-effective at different willingness-to-pay thresholds based on 100,000 iterations for a simulated cohort of 100,000 patients.
